# Smoke-free legislation and child health

**DOI:** 10.1038/npjpcrm.2016.67

**Published:** 2016-11-17

**Authors:** Timor Faber, Jasper V Been, Irwin K Reiss, Johan P Mackenbach, Aziz Sheikh

**Affiliations:** 1 Division of Neonatology, Department of Paediatrics, Erasmus University Medical Centre—Sophia Children’s Hospital, Rotterdam, The Netherlands; 2 Department of Public Health, Erasmus University Medical Centre, Rotterdam, The Netherlands; 3 School for Public Health and Primary Care (CAPHRI), Maastricht University Medical Centre, Maastricht, The Netherlands; 4 Centre of Medical Informatics, Usher Institute of Population Health Sciences and Informatics, The University of Edinburgh, Edinburgh, UK; 5 Division of General Internal Medicine and Primary Care, Brigham and Women’s Hospital/Harvard Medical School, Boston, MA, USA; 6 Department of Medicine, Harvard Medical School, Boston, MA, USA

## Abstract

In this paper, we aim to present an overview of the scientific literature on the link between smoke-free legislation and early-life health outcomes. Exposure to second-hand smoke is responsible for an estimated 166 ,000 child deaths each year worldwide. To protect people from tobacco smoke, the World Health Organization recommends the implementation of comprehensive smoke-free legislation that prohibits smoking in all public indoor spaces, including workplaces, bars and restaurants. The implementation of such legislation has been found to reduce tobacco smoke exposure, encourage people to quit smoking and improve adult health outcomes. There is an increasing body of evidence that shows that children also experience health benefits after implementation of smoke-free legislation. In addition to protecting children from tobacco smoke in public, the link between smoke-free legislation and improved child health is likely to be mediated via a decline in smoking during pregnancy and reduced exposure in the home environment. Recent studies have found that the implementation of smoke-free legislation is associated with a substantial decrease in the number of perinatal deaths, preterm births and hospital attendance for respiratory tract infections and asthma in children, although such benefits are not found in each study. With over 80% of the world’s population currently unprotected by comprehensive smoke-free laws, protecting (unborn) children from the adverse impact of tobacco smoking and SHS exposure holds great potential to benefit public health and should therefore be a key priority for policymakers and health workers alike.

## Introduction

Despite the fact that the adverse health effects of tobacco smoking were officially recognised over 50 years ago, smoking continues to be the leading cause of preventable death worldwide.^1^ Globally, over one billion people are regular smokers, and annually an estimated six million people die as a consequence of smoking.^2^ The prevalence of smoking is highest in the reproductive age range, and approximately 10–20% of women in high-income countries smoke throughout pregnancy.^3^ Smoking during pregnancy causes long-lasting epigenetic changes,^4^ increases the risk for adverse health outcomes during fetal life and childhood^1^ and may have long-term consequences in later life, even for subsequent generations.^5^

Besides active smoking, exposure to second-hand smoke (SHS) is responsible for an estimated 600,000 deaths and almost 11 million disability-adjusted life-years per year worldwide.^6^ Maternal SHS exposure during pregnancy is associated with increased risks for stillbirth, low birthweight and paediatric asthma.^1,7–10^ After birth, an estimated 40–50% of the world’s children are regularly exposed to SHS, primarily by being around smoking parents and/or other household members.^6,11^ As a result, children make up over a quarter of all deaths and over half of all disability-adjusted life-years associated with SHS exposure.^6^ These estimates are based on the impact of SHS on respiratory tract infections (RTIs) and asthma only, and the estimated burden of death and disease is likely much larger when in addition considering other SHS-associated outcomes including adverse perinatal outcomes, sudden infant death syndrome (SIDS) and invasive meningococcal infections.^6,12–14^

**Figure 1:**
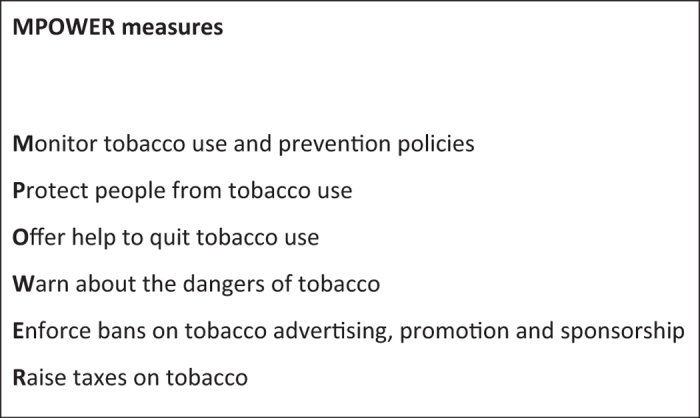
MPOWER measures. MPOWER is an acronym of the six categories of tobacco control measures recommended by the World Health Organisation to combat the death and disease burden caused by smoking and exposure to second-hand smoke. Source: WHO report on the global tobacco epidemic, 2015: raising taxes on tobacco.^2^

To avoid this preventable burden of death and disease from smoking and exposure to SHS, the World Health Organization (WHO) established the international FCTC (Framework Convention on Tobacco Control) in 2005.^2^ Having been ratified by 180 countries, this treaty currently covers more than 90% of the world’s population.^15^ To support participating countries in implementing the agreements within the FCTC, the WHO introduced a set of six policy measures in 2008 (represented by the MPOWER acronym—Figure 1).^2^ As part of the MPOWER measures, the WHO urges all countries to protect their people from exposure to SHS by introducing comprehensive smoke-free legislation to prohibit smoking in all indoor public spaces, including workplaces.^2^ Besides reductions in SHS exposure, smoke-free laws have also been associated with decreases in smoking prevalence and in young people taking up smoking.^16,17^ There is also consistent evidence for an association between smoke-free legislation and reductions in hospital admissions and deaths owing to tobacco-associated cardiovascular and respiratory diseases among adults.^18,19^ As developing humans, children are particularly vulnerable to tobacco smoke and generally cannot control their own exposure to tobacco smoke. It is therefore important to consider the potential benefits of smoke-free legislation to child health via reducing both antenatal and postnatal SHS exposure.

## Aim

The aim of this paper is to present an overview of the scientific literature on the link between smoke-free legislation and perinatal and paediatric health outcomes.

## Identification of papers

We performed a semi-structured search in PubMed to identify recently published studies from 2005 up to June 2016, using the following search equation: ‘(smok* OR tobacco OR cigar*) AND (legislation* OR policy OR policies OR ban OR bans OR law OR laws) AND (fetus OR fetal OR stillbirth OR newborn OR neonatal OR baby OR babies OR infant OR child)'. We searched for studies that examined the association between the introduction of smoke-free legislation and health-related outcomes among (unborn) children.

## Studying the link between smoke-free legislation and child health

A randomised controlled trial is generally considered the optimal design to study the effectiveness of health interventions. National public health policies such as the implementation of smoke-free legislation are, however, generally not amendable to being implemented in a randomised fashion. In such situations, it is therefore necessary to consider alternative options to make causal inferences regarding the relationship between an intervention and health outcomes.^20,21^

Quasi-experimental studies, such as controlled before–after studies and interrupted time series studies, can provide a robust alternative to randomised controlled trials in such situations and have been used to assess the potential population health benefits of smoke-free legislation.^22^ Assessing causality from such studies is, however, difficult owing to the inherent risk of bias and confounding. Studies should therefore be carefully designed, and in attempting to make causal inferences, it is (among other things) important to develop logic models of how the intervention might work and what process measures might help to shed light on the mechanisms of action, the effect size and consistency of the evidence.^23^

## Smoke-free legislation and child health: likely causal pathways

**Figure 2:**
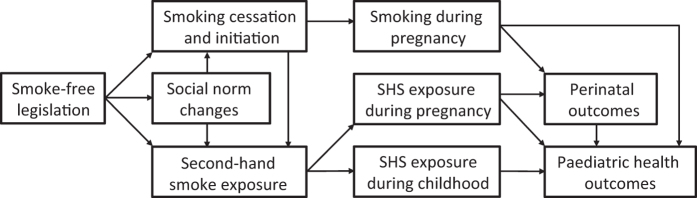
Likely causal pathways between smoke-free legislation and perinatal and paediatric health outcomes. Source: Adapted from Peelen *et al*.^24^

One may argue that children are unlikely to be sensitive to a policy change primarily aimed at reducing SHS exposure in public places such as the workplace, bars and restaurants. Findings from several studies, however, provide support for a number of pathways underlying a potential causal link between smoke-free legislation and improved child health. We have summarised these in a directed acyclic graph (Figure 2), and we discuss the supporting evidence below, by timing of tobacco smoke exposure: antenatal versus postnatal.

### Maternal smoking, SHS exposure and adverse pregnancy outcomes

When a pregnant woman smokes or is exposed to SHS, many constituents of tobacco smoke readily cross the placenta and expose the developing fetus to the associated health risks.^25^ Active maternal smoking during pregnancy is a recognised risk factor for a variety of adverse pregnancy outcomes, including stillbirth,^7^ low birthweight,^1^ preterm birth,^26^ small for gestational age (SGA) birth,^27^ congenital anomalies^28^ and neonatal mortality.^1^ It is, in addition, associated with adverse health outcomes during childhood, including SIDS,^14^ wheezing/asthma^29,30^ and RTIs (Table 1).^32^ Smoking cessation during pregnancy has been shown to normalise many of these risks.^34–36^

Similar to active smoking, SHS exposure during pregnancy is associated with increased risks of stillbirth,^8^ low birthweight,^9^ congenital anomalies^8^ and development of childhood wheezing/asthma (Table 1).^30^ Although very few studies have evaluated the effectiveness of interventions to reduce SHS exposure during pregnancy,^37^ it appears that such interventions have the potential to improve birth outcomes, which include reducing the incidence of very low birthweight and very preterm birth.^38^ This suggests that, similar to the perinatal health risks associated with active smoking during pregnancy, those related to SHS exposure are indeed avoidable.

### Smoke-free legislation and maternal smoking and SHS exposure

A number of studies support a link between the implementation of smoke-free laws and a reduction in maternal smoking during pregnancy.^39–44^ In Scotland, for example, the prevalence of maternal smoking during pregnancy decreased from 25.4 to 18.8% (*P*<0.001) after the 2006 implementation of comprehensive legislation prohibiting smoking in public places.^42^ Improved perinatal outcomes, namely decrease in low birthweight and preterm delivery, were found among both women who smoked and those who did not smoke during pregnancy.^42^ This suggests that reductions in both active smoking and SHS exposure during pregnancy contribute to the observed drop in adverse perinatal outcomes sensitive to tobacco smoke.^41,42,45^

Although we are unaware of studies assessing the association between smoke-free legislation and SHS exposure during pregnancy specifically, significant reductions in SHS exposure among the general adult population were observed in the majority of studies on the topic.^16,18^ Much of this effect is likely owing to the actual smoking restrictions in public places. Many studies have, in addition, demonstrated a drop in smoking initiation among the youth,^17,46^ a drop in overall smoking prevalence and cigarette consumption and an increase in smoking cessation after the introduction of smoke-free legislation, likely contributing to the overall reduction in SHS exposure (Figure 2).^16^

### SHS exposure and child health

Children’s exposure to SHS is associated with SIDS,^14^ wheezing/asthma,^29,30,47^ RTIs,^32^ otitis media with effusion^48^ and meningococcal disease.^13^ (Table 2) These risks have been shown to be independent of those associated with maternal smoking during pregnancy.^14,30,32,48^

### Smoke-free legislation and childhood SHS exposure

Smoke-free legislation has been associated with reductions in SHS exposure among children in various countries.^49–52^ Although smoke-free legislation is primarily aimed at reducing SHS exposure in public places, the primary location for children to be exposed is the home environment.^53,54^ In this context, it is important to note that, in addition to reducing SHS exposure in public places, implementation of smoke-free legislation is also associated with reductions in smoking at home and with increased adoption of home-smoking bans.^53–57^ This illustrates how, through norm spreading, smoke-free legislation may carry additional benefits mediated via reducing SHS exposure beyond locations primarily targeted.

## Smoke-free legislation and early-life health outcomes

Through the previously described changes in maternal smoking and antenatal and postnatal SHS exposure after the implementation of smoke-free legislation, one might expect to also observe changes in smoking- and SHS-associated perinatal and paediatric health outcomes. Below we discuss evidence from studies assessing the link between smoke-free legislation and perinatal and child health (summarised in Table 3).

### Perinatal health outcomes

#### Perinatal mortality

Consistent with the recognised link between antenatal smoke exposure and perinatal mortality, the introduction of a comprehensive smoke-free law in England was associated with an immediate −7.8% (95% confidence interval (95% CI): −11.8 to −3.5) reduction in stillbirths and a −7.6% (−11.7 to −3.4) reduction in neonatal deaths.^45^ We estimated that, per year, over 240 stillbirths and more than 100 neonatal deaths have been averted across England since the implementation of the legislation.^45^ In a smaller study in the Netherlands, we did not observe a significant association between phased introduction of smoke-free legislation, introduced in conjunction with other tobacco control policies, and perinatal mortality.^24^ We speculated that lack of enforcement and lower compliance compared with that in England may have attenuated the impact of smoke-free legislation on these outcomes in the Netherlands.^63–67^ Other factors including methodological and population differences may also have contributed to the discrepant findings, and additional studies are needed to investigate the impact of comprehensive smoke-free legislation on perinatal mortality.

#### Low birthweight

In a systematic review published in 2014,^58^ we identified six studies assessing the association between smoke-free legislation and low birthweight.^41,42,44,68–70^ One study demonstrated an immediate reduction in low birthweight,^42^ whereas another showed a gradual reduction.^70^ In a meta-analysis combining all six studies, however, neither the immediate nor the gradual reduction was statistically significant.^58^ Follow-on studies also show mixed evidence.^24,40,45,59,71,72^ A closer look at this evidence suggests that reductions in low birthweight are generally observed in countries with the most comprehensive laws.^40,42,45,59^

Low birthweight is usually the result of either short gestation (preterm birth) or fetal growth restriction (SGA), and a number of studies have evaluated the association between smoke-free legislation and these outcomes.

#### Preterm birth

In our systematic review on smoke-free legislation and child health, we identified four studies investigating the association with preterm birth.^41,42,44,69^ Three demonstrated a statistically significant decrease in preterm birth,^42,44,69^ resulting in a pooled reduction of −10.4% (95% CI: −18.8 to −2.0) in meta-analysis.^58^ The association between smoke-free legislation and a drop in preterm birth was later confirmed in Quebec.^59^ A recent Swiss study provided evidence of a ‘dose–response’ relationship, with the largest reductions in preterm births observed among pregnancies that were fully protected by smoke-free legislation (versus only from the second or third trimester) and in areas with the most comprehensive legislation.^73^ Furthermore, a reduction in very preterm births was observed following extension of the smoke-free workplace law in the Netherlands to include hospitality venues.^24^ On the other hand, two large evaluations of local US smoke-free laws failed to demonstrate a link between smoke-free laws and preterm birth.^71,72^ A systematic and comprehensive assessment of the strengths and weaknesses of these studies, and the coverage and enforcement of and compliance with the legislation in relation to the reported effect sizes, is ongoing and will help to better understand the discrepant findings.^74^

#### Small for gestational age

Being SGA is a more specific indicator of intrauterine growth restriction than is low birthweight, as it takes into account the gestational age of the baby. Several studies support a link between smoke-free legislation and a reduction in the risk of babies being born SGA. In a meta-analysis of three studies, although the overall reduction in SGA babies was not statistically significant (−1.40%, 95% CI: −3.20 to 0.40), the number of very SGA babies dropped by −5.30% (95% CI: −5.42 to −5.18).^58^ Follow-on work conducted in Quebec and the Netherlands also showed significant reductions in the risk for SGA birth following smoke-free legislation.^24,59^ Again, this was mainly owing to a reduction in very SGA births.^24^

### Child health outcomes

#### Infant mortality

In our analysis of the smoke-free law in England, we observed an immediate reduction in infant mortality (−6.3%, 95% CI: −9.6 to −2.9) following its implementation.^45^ This was mainly attributable to the reduction in neonatal deaths described earlier.^45^ Despite the strong epidemiological link between SHS exposure and SIDS, we did not, however, observe a significant change in the odds of SIDS in this study. Although an ecological study in the United States did demonstrate an association between strong smoke-free policies and reduced SIDS incidence,^75^ this was not confirmed in a multi-country analysis conducted by the same researchers.^76^ Thus, although smoke-free legislation appears to be associated with an overall reduction in infant mortality,^45^ this cannot be attributed to a reduction in SIDS.

#### Asthma

Asthma remains the most common chronic disease in childhood,^77^ and childhood asthma is linked to the development of long-term reduced lung function and chronic obstructive pulmonary disease.^78^ Consistent evidence supports an association between smoke-free legislation and a reduction in childhood hospital attendance for asthma exacerbations. In our systematic review, we identified four studies evaluating this link.^79–82^ In a meta-analysis, the implementation of smoke-free legislation was associated with a −10.1% (95% CI: −15.2 to −5.0) decrease in paediatric emergency department visits and hospital admissions for asthma.^58^ This association was confirmed in later studies conducted in the United States,^62,83,84^ and is in line with meta-analyses of adult studies demonstrating a link between smoke-free legislation and a reduction in severe respiratory events (i.e., deaths or hospital admissions).^19^ Other studies show that smoke-free policies do not affect overall asthma incidence among children, suggesting that the observed reductions in hospital attendance are indeed the result of a drop in asthma exacerbations.^85,86^

#### Respiratory tract infections

RTIs account for the majority of the disease burden associated with SHS exposure among children.^6^ In our 2014 systematic review, we did not, however, identify any studies that investigated the association between the implementation of smoke-free legislation and RTIs.^58^ We addressed this in an evaluation of the 2007 comprehensive smoke-free law in England, using data of over 1.6 million paediatric hospital admissions for RTIs over a 12-year period. There was an immediate −3.5% (95% CI: −4.7 to −2.3) decrease in RTI admissions, which was mainly attributable to a reduction in lower RTI admissions (−13.8% (95% CI: −15.6 to −12.0)).^60^ This translated into an estimated ~11,000 paediatric RTI hospital admissions being averted per year during the first 5 years following the implementation of smoke-free legislation. This link between smoke-free legislation and a reduction in hospital attendance for paediatric lower RTIs was recently confirmed in an evaluation of the 2007 comprehensive smoke-free law in Hong Kong,^61^ and a multi-state analysis in the United States.^62^ Similar to the studies on asthma, although hospital admissions for RTIs went down after smoke-free legislation,^60–62^ there did not appear to have been an overall reduction in RTI incidence.^85^ The observed drops in RTI hospitalisations are therefore more likely to represent reductions in disease severity rather than incidence, consistent with epidemiological evidence identifying SHS as a risk factor for severe RTI presentations among children.^87,88^

### Smoke-free legislation and health inequalities

Tobacco smoking and SHS exposure are important drivers of socio-economic disparities in health outcomes and life expectancy. There are concerns regarding the potential for certain tobacco control policies to have a negative equity impact.^89^ In England, the reduction in paediatric RTI admissions following the smoke-free law was largest among children from deprived areas, suggesting that a pro-equity effect may be present.^60^ There was, however, no differential association between smoke-free legislation and child health according to socio-economic status in other studies having evaluated this.^59,72,80^

## Interpreting the evidence

Clearly over recent years, the evidence base assessing the potential links between smoke-free legislation and population health, including that of children, has grown substantially. Interpretation of the totality of evidence is, however, complicated not only by the fact that it is derived from quasi-experimental evaluations but also by the range of additional potential sources of variation between studies. Examples include geographical location, population demographics, comprehensiveness and enforcement of the legislation, public support for and compliance with the law, degree of associated social norm spreading and methodological aspects of the studies, including choice of statistical models and approaches to handling sources of bias and potential confounding. Such factors may have contributed to some of the observed inconsistencies between findings of individual studies. Meta-epidemiological evaluations of studies assessing health outcomes following smoke-free legislation among adults have already allowed some of this variation to be taken into account when interpreting the evidence.^19^ With the number of studies in the paediatric field continuing to increase, similar opportunities now open up to further increase confidence in the conclusions made according to these studies.^74^

## Implications for policy and practice

Despite the limitations of the existing evidence base, it provides considerable support for a link between smoke-free legislation and improved perinatal and child health outcomes. Reductions in maternal smoking during pregnancy and in SHS exposure during pregnancy and childhood are likely to be the main drivers behind the observed reductions in adverse early-life health outcomes (Figure 2). The observed associations are in line with the recognised causal links between prenatal and childhood tobacco smoke exposure and adverse perinatal and paediatric health outcomes, as well as the already recognised substantial population health effects of smoke-free policies among adults.^16,18,19,58^ Improved compliance with WHO guidelines to address the ongoing global tobacco epidemic and reduce its associated substantial burden of death and disease is clearly needed so that more and more (unborn) children as well as adults can enjoy these health benefits. Over 80% of the world’s population, however, currently remains unprotected by comprehensive smoke-free laws, and global compliance with other MPOWER policies is smaller still.^2^ Smoke-free laws are generally well supported by the public and appear highly cost-effective.^90,91^ It is crucial for policymakers to realise that such policies are most effective in reducing SHS exposure and improving population health when implemented in a comprehensive manner (i.e., covering both workplaces and the hospitality industry).^19,40,92^ Smoke-free laws should ideally be part of an integrated tobacco endgame strategy in which, among other aspects, tobacco tax increase is an important element.^93^ Tobacco taxes are considered the most effective measure to reduce tobacco use,^2^ and have been demonstrated to also benefit perinatal and infant health via reductions in maternal smoking rates during pregnancy.^39,72,76,94,95^

Protecting (unborn) children from the adverse impact of tobacco smoking and SHS exposure should be a key priority for policymakers and health workers alike. Health practitioners have a responsibility to discuss the dangers of active smoking and second- and third-hand smoke exposure (i.e., exposure to toxic tobacco constituents via clothing, curtains, carpets, etc.) in relation to children’s health with relevant family members and pre-conceptional and pregnant women. They should be aware of the existing local care pathways to support smoking cessation and be ready to offer cessation advice and pharmacological support when necessary.

## Knowledge gaps and future research

Now that several health benefits of comprehensive legislation to prohibit smoking in indoor public places have been established, there is a need to evaluate the effectiveness of reducing SHS exposure in other places. An increasing number of countries are now implementing laws to protect children from SHS in outdoor areas (e.g., playgrounds, school grounds, parks, beaches) and private environments, such as cars.^2,96–99^ There is a need to evaluate the effectiveness of such policies in reducing SHS exposure, denormalising smoking and improving child health to inform policymakers in other countries that may be considering implementing similar legislation. There is furthermore a need to identify the effectiveness of additional policy strategies, such as tobacco taxes and mass media campaigns, and combinations of such policies to, in particular, improve child health and reduce smoking initiation among children. Ongoing work to collate existing studies on tobacco control policies and child health via a systematic review will help provide a comprehensive assessment of the available evidence on this topic.^74^ Given the shifting burden of smoking and tobacco-related death and disease from high-income to low- and middle-income countries, there is a particular need to assess the impact of tobacco control strategies in such countries and develop tools to support their policymakers in prioritising the most effective policies to improve population health.^58^ In parallel to such policy-based evaluations, researchers should continue to develop, evaluate and help implement strategies and interventions to prevent SHS exposure among children at the individual, family and community level.

### Conclusions

Evidence suggests that comprehensive smoke-free legislation is associated with reductions in perinatal mortality, preterm birth and paediatric hospital admissions for RTIs and asthma.^45,58,60,61^ There is a clear need for wider implementation of comprehensive smoke-free policies across the globe as a key constituent of tobacco endgame strategies in accordance with FCTC recommendations to improve the population health of (unborn) children and adults alike.^2^

## Figures and Tables

**Table 1:** Selected child health outcomes associated with antenatal tobacco smoke exposure

*Outcome*	*Effect size (95% CI)*
*Maternal smoking during pregnancy*	
Perinatal outcomes	
Stillbirth	1.4 (1.27–1.46); ref. 7
Preterm birth	1.27 (1.21–1.33); ref. 26
Birth defects (gastrointestinal)	1.27 (1.18–1.35); ref. 28
Birth defects (cardiovascular)	1.09 (1.02–1.17); ref. 28
Birth defects (musculoskeletal)	1.16 (1.05–1.27); ref. 28
Birth defects (central nervous system)	1.10 (1.01–1.19); ref. 28
Childhood outcomes	
Sudden infant death syndrome	2.25 (2.03–2.50); ref. 14
Early wheezing (age ⩽2 years)	1.33 (1.03–1.71); ref. 30
Recurrent wheezing	1.49 (1.33–1.67); ref. 31
Wheezing/asthma in ⩾6-year-olds	1.22 (1.03–1.44); ref. 29
Lower respiratory infections	1.24 (1.11–1.38); ref. 32
Overweight	1.33 (1.23–1.44); ref. 33
Obesity	1.60 (1.37–1.88); ref. 33
	
*Maternal second-hand smoke exposure during pregnancy*	
Perinatal outcomes	
Stillbirth	1.23 (1.09–1.38); ref. 8
Low birthweight	1.32 (1.07–1.63); ref. 9
Birth defects	1.13 (1.01–1.26); ref. 8
Childhood outcomes	
Early wheezing (age ⩽2 years)	1.11 (1.03–1.20); ref. 30

Outcomes with positive associations demonstrated in meta-analyses are shown.

Abbreviation: CI, confidence interval.

**Table 2:** Selected child health outcomes associated with postnatal tobacco smoke exposure

*Outcome*	*Effect size (95% CI)*
Sudden infant death syndrome	1.97 (1.77–2.19); ref. 14
Early wheezing (age ⩽2 years)	1.29 (1.19–1.40); ref. 30
Wheezing/asthma in ⩾6-year-olds	1.30 (1.13–1.51); ref. 29
Hospitalisation for asthma exacerbation	1.85 (1.20–2.86); ref. 47
Lower respiratory infections	1.54 (1.40–1.69); ref. 32
Middle ear infection (including otitis media with effusion)	1.32 (1.20–1.45); ref. 48
Meningococcal disease	2.02 (1.52–2.69); ref. 13

Outcomes with positive associations demonstrated in meta-analyses are shown.

Abbreviation: CI, confidence interval.

**Table 3:** Association between smoke-free legislation and changes in child health outcomes

*Outcome*	*Effect size (95% CI)*	*Study type*	*Location(s)*	*Legislation comprehensive?*
*Perinatal outcomes*				
Stillbirth	−7.8% (−11.8 to −3.5); ref. 45	ITS	England	Yes
	−1% (−9 to 8)/−3% (−12 to 6); ref. 24	ITS	Netherlands	No
Low birthweight	−1.7% (−5.1 to 1.6); ref. 58	MA	Belgium, Norway, Scotland, USA	Mixed
Very low birthweight	−35.4% (−111.1 to 40.3); ref. 58	MA	Norway, USA	No
Preterm birth	−10.4% (−18.8 to −2.0); ref. 58	MA	Belgium, Norway, Scotland, USA	Mixed
Very preterm birth	−17.4% (−26.9 to −6.7); ref. 42	ITS	Scotland	Yes
	−2.3%^a^ (−3.7 to −0.9); ref. 59	ITS	Quebec, Canada	Yes
	−6% (−14 to 3)/−11% (−19 to −3); ref. 24	ITS	Netherlands	No
SGA	−1.4% (−3.2 to 0.4); ref. 58	MA	Belgium, Ireland, Scotland	Mixed
Very SGA	−5.3% (−5.4 to −5.2); ref. 58	MA	Ireland, Scotland	Yes
Congenital anomalies	1% (−6 to 8)/−2% (−9 to 6); ref. 24	ITS	Netherlands	No
	−0.03%^a^ (−4.0 to 3.9); ref. 41	CITS	Norway	No
Neonatal mortality	−7.6% (−11.7 to −3.4); ref. 45	ITS	England	Yes
	−3% (−16 to 12)/−12% (−24 to 2); ref. 24	ITS	Netherlands	No
				
*Childhood outcomes*				
Infant mortality	−6.3% (−9.6 to −2.9); ref. 45	ITS	England	Yes
Sudden infant death syndrome	1.8% (−8.4 to 13.2); ref. 45	ITS	England	Yes
Asthma hospital attendance	−10.1% (−15.2 to −5.0); ref. 58	MA	Canada, England, USA	Mixed
RTI hospital admissions	−3.5% (−4.5 to −2.3); ref. 60	ITS	England	Yes
Lower RTI hospital admissions	−13.8% (−15.6 to −12.0); ref. 60	ITS	England	Yes
	−33.5% (−36.4 to −30.5); ref. 61	ITS	Hong Kong	Yes
Lower RTI emergency department visits	−8% (−13 to −4%); ref. 62	CITS	USA	Mixed

For each outcome, findings from meta-analysis are shown where available; findings from individual studies are shown otherwise. For the study conducted in the Netherlands, figures represent the impact of smoke-free legislation in the workplace, and extension to bars and restaurants, respectively.

Abbreviations: CI, confidence interval; CITS, interrupted time series with control group; ITS, interrupted time series; MA, meta-analysis; RTI, respiratory tract infection; SGA, small for gestational age.

a Absolute change (percentage points).

## References

[bib1] National Center for Chronic Disease Prevention and Health Promotion, Office on Smoking and Health. The Health Consequences of Smoking—50 Years of Progress: A Report of the Surgeon General (U.S. Department of Health and Human Services, Centers for Disease Control and Prevention: Atlanta, GA, USA, 2014).

[bib2] WHO Report on the Global Tobacco Epidemic, 2015: Raising Taxes on Tobacco. (World Health Organization, 2015).

[bib3] Miyazaki, Y., Hayashi, K. & Imazeki, S. Smoking cessation in pregnancy: psychosocial interventions and patient-focused perspectives. Int. J. Womens Health 7, 415–427 (2015). 25960677 10.2147/IJWH.S54599 PMC4411022

[bib4] Joubert, B. R. et al. DNA methylation in newborns and maternal smoking in pregnancy: genome-wide consortium meta-analysis. Am. J. Hum. Genet. 98, 680–696 (2016). 27040690 10.1016/j.ajhg.2016.02.019 PMC4833289

[bib5] Bao, W. et al. Parental smoking during pregnancy and the risk of gestational diabetes in the daughter. Int. J. Epidemiol. 45, 160–169 (2016). 26748845 10.1093/ije/dyv334 PMC4881834

[bib6] Oberg, M., Jaakkola, M. S., Woodward, A., Peruga, A. & Pruss-Ustun, A. Worldwide burden of disease from exposure to second-hand smoke: a retrospective analysis of data from 192 countries. Lancet 377, 139–146 (2011). 21112082 10.1016/S0140-6736(10)61388-8

[bib7] Flenady, V. et al. Major risk factors for stillbirth in high-income countries: a systematic review and meta-analysis. Lancet 377, 1331–1340 (2011). 21496916 10.1016/S0140-6736(10)62233-7

[bib8] Leonardi-Bee, J., Britton, J. & Venn, A. Secondhand smoke and adverse fetal outcomes in nonsmoking pregnant women: a meta-analysis. Pediatrics 127, 734–741 (2011). 21382949 10.1542/peds.2010-3041

[bib9] Leonardi-Bee, J., Smyth, A., Britton, J. & Coleman, T. Environmental tobacco smoke and fetal health: systematic review and meta-analysis. Arch. Dis. Child. Fetal Neonatal Ed. 93, F351–F361 (2008). 18218658 10.1136/adc.2007.133553

[bib10] Simons, E., To, T., Moineddin, R., Stieb, D. & Dell, S. D. Maternal second-hand smoke exposure in pregnancy is associated with childhood asthma development. J. Allergy Clin. Immunol. Pract. 2, 201–207 (2014). 24607049 10.1016/j.jaip.2013.11.014

[bib11] Mbulo L. et al. Secondhand smoke exposure at home among one billion children in 21 countries: findings from the Global Adult Tobacco Survey (GATS). Tob. Control. http://dx.doi.org/10.1136/tobaccocontrol-2015-052693 (2016). 10.1136/tobaccocontrol-2015-052693 PMC5488799 26869598

[bib12] Blencowe, H. et al. National, regional, and worldwide estimates of preterm birth rates in the year 2010 with time trends since 1990 for selected countries: a systematic analysis and implications. Lancet 379, 2162–2172 (2012). 22682464 10.1016/S0140-6736(12)60820-4

[bib13] Lee, C. C., Middaugh, N. A., Howie, S. R. & Ezzati, M. Association of secondhand smoke exposure with pediatric invasive bacterial disease and bacterial carriage: a systematic review and meta-analysis. PLoS Med. 7, e1000374 (2010). 21151890 10.1371/journal.pmed.1000374 PMC2998445

[bib14] Zhang, K. & Wang, X. Maternal smoking and increased risk of sudden infant death syndrome: a meta-analysis. Leg Med (Tokyo) 15, 115–121 (2013). 23219585 10.1016/j.legalmed.2012.10.007

[bib15] United Nations. United Nations Treaty Collection: Chapter IX: Health 4. WHO Framework Convention on Tobacco Control [Internet]. Geneva: United Nations; 21 May 2003 (updated 7 April 2016; cited 7 April 2016). Available from https://treaties.un.org/pages/ViewDetails.aspx?src=TREATY&mtdsg_no=IX-4&chapter=9&lang=en.

[bib16] Hoffman, S. J. & Tan, C. Overview of systematic reviews on the health-related effects of government tobacco control policies. BMC Public Health 15, 744 (2015). 26242915 10.1186/s12889-015-2041-6 PMC4526291

[bib17] Katikireddi, S. V., Der, G., Roberts, C. & Haw, S. Has childhood smoking reduced following smoke-free public places legislation? A segmented regression analysis of cross-sectional UK school-based surveys. Nicotine Tob. Res. http://dx.doi.org/10.1093/ntr/ntw018 (2016). 10.1093/ntr/ntw018 PMC4902887 26911840

[bib18] Frazer, K. et al. Legislative smoking bans for reducing harms from secondhand smoke exposure, smoking prevalence and tobacco consumption. Cochrane Database Syst. Rev. 2, CD005992 (2016). 26842828 10.1002/14651858.CD005992.pub3 PMC6486282

[bib19] Tan, C. E. & Glantz, S. A. Association between smoke-free legislation and hospitalizations for cardiac, cerebrovascular, and respiratory diseases: a meta-analysis. Circulation 126, 2177–2183 (2012). 23109514 10.1161/CIRCULATIONAHA.112.121301 PMC3501404

[bib20] Biglan, A., Ary, D. & Wagenaar, A. C. The value of interrupted time-series experiments for community intervention research. Prev. Sci. 1, 31–49 (2000). 11507793 10.1023/a:1010024016308 PMC4553062

[bib21] Kontopantelis, E., Doran, T., Springate, D. A., Buchan, I. & Reeves, D. Regression based quasi-experimental approach when randomisation is not an option: interrupted time series analysis. BMJ 350, h2750 (2015). 26058820 10.1136/bmj.h2750 PMC4460815

[bib22] Effective Practice and Organisation of Care (EPOC). What Study Designs Should Be Included in an EPOC Review? EPOC Resources for Review Authors [Internet] (Norwegian Knowledge Centre for the Health Services, 2013). Available from: http://epoc.cochrane.org/epoc430specific-resources-review-authors.

[bib23] Been, J. V. & Sheikh, A. Investigating the link between smoke-free legislation and stillbirths. Expert Rev. Respir. Med. 10, 109–112 (2016). 26610241 10.1586/17476348.2016.1125784

[bib24] Peelen, M. J. et al. Tobacco control policies and perinatal health: a national quasiexperimental study. Sci. Rep. 6, 23907 (2016). 27103591 10.1038/srep23907 PMC4840332

[bib25] Wagijo, M. A., Sheikh, A., Duijts, L. & Been, J. V. Reducing tobacco smoking and smoke exposure to prevent preterm birth and its complications. Paediatr. Respir. Rev. http://dx.doi.org/10.1016/j.prrv.2015.09.002 (2015). 10.1016/j.prrv.2015.09.002 26482273

[bib26] Shah, N. R. & Bracken, M. B. A systematic review and meta-analysis of prospective studies on the association between maternal cigarette smoking and preterm delivery. Am. J. Obstet. Gynecol. 182, 465–472 (2000). 10694353 10.1016/s0002-9378(00)70240-7 PMC2706697

[bib27] Ko, T. J. et al. Parental smoking during pregnancy and its association with low birth weight, small for gestational age, and preterm birth offspring: a birth cohort study. Pediatr. Neonatol. 55, 20–27 (2014). 23850094 10.1016/j.pedneo.2013.05.005

[bib28] Hackshaw, A., Rodeck, C. & Boniface, S. Maternal smoking in pregnancy and birth defects: a systematic review based on 173 687 malformed cases and 11.7 million controls. Hum. Reprod. Update 17, 589–604 (2011). 21747128 10.1093/humupd/dmr022 PMC3156888

[bib29] Silvestri, M., Franchi, S., Pistorio, A., Petecchia, L. & Rusconi, F. Smoke exposure, wheezing, and asthma development: a systematic review and meta-analysis in unselected birth cohorts. Pediatr. Pulmonol. 50, 353–362 (2015). 24648197 10.1002/ppul.23037

[bib30] Vardavas C. I. et al. The independent role of prenatal and postnatal exposure to active and passive smoking on the development of early wheeze in children. Eur. Respir. J. 2016; 48: 115–124. 26965294 10.1183/13993003.01016-2015

[bib31] Duan, C. et al. Association between maternal smoking during pregnancy and recurrent wheezing in infancy: evidence from a meta-analysis. Int. J. Clin. Exp. Med. 8, 6755–6761 (2015). 26221213 PMC4509158

[bib32] Jones, L. L. et al. Parental and household smoking and the increased risk of bronchitis, bronchiolitis and other lower respiratory infections in infancy: systematic review and meta-analysis. Respir. Res. 12, 5 (2011). 21219618 10.1186/1465-9921-12-5 PMC3022703

[bib33] Riedel, C. et al. Parental smoking and childhood obesity: higher effect estimates for maternal smoking in pregnancy compared with paternal smoking—a meta-analysis. Int. J. Epidemiol. 43, 1593–1606 (2014). 25080528 10.1093/ije/dyu150

[bib34] McCowan, L. M. et al. Spontaneous preterm birth and small for gestational age infants in women who stop smoking early in pregnancy: prospective cohort study. BMJ 338, b1081 (2009). 19325177 10.1136/bmj.b1081 PMC2661373

[bib35] Suzuki, K. et al. Effect of maternal smoking cessation before and during early pregnancy on fetal and childhood growth. J. Epidemiol. 24, 60–66 (2014). 24335086 10.2188/jea.JE20130083 PMC3872526

[bib36] Vardavas, C. I. et al. Smoking and smoking cessation during early pregnancy and its effect on adverse pregnancy outcomes and fetal growth. Eur. J. Pediatr. 169, 741–748 (2010). 19953266 10.1007/s00431-009-1107-9

[bib37] Tong, V. T. et al. Clinical interventions to reduce secondhand smoke exposure among pregnant women: a systematic review. Tob. Control 24, 217–223 (2015). 24789602 10.1136/tobaccocontrol-2013-051200 PMC4924528

[bib38] El-Mohandes, A. A., Kiely, M., Blake, S. M., Gantz, M. G. & El-Khorazaty, M. N. An intervention to reduce environmental tobacco smoke exposure improves pregnancy outcomes. Pediatrics 125, 721–728 (2010). 20211945 10.1542/peds.2009-1809 PMC2923806

[bib39] Adams, E. K. et al. Reducing prenatal smoking: the role of state policies. Am. J. Prev. Med. 43, 34–40 (2012). 22704743 10.1016/j.amepre.2012.02.030

[bib40] Bartholomew, K. S. & Abouk, R. The effect of local smokefree regulations on birth outcomes and prenatal smoking. Matern. Child Health J. 20, 1526–1538 (2016). 26987859 10.1007/s10995-016-1952-x

[bib41] Bharadwaj, P., Johnsen, J. V. & Løken, K. V. Smoking bans, maternal smoking and birth outcomes. J. Public Economics 115, 72–93 (2014).

[bib42] Mackay, D. F., Nelson, S. M., Haw, S. J. & Pell, J. P. Impact of Scotland's smoke-free legislation on pregnancy complications: retrospective cohort study. PLoS Med. 9, e1001175 (2012). 22412353 10.1371/journal.pmed.1001175 PMC3295815

[bib43] Nguyen, K. H., Wright, R. J., Sorensen, G. & Subramanian, S. V. Association between local indoor smoking ordinances in Massachusetts and cigarette smoking during pregnancy: a multilevel analysis. Tob. Control 22, 184–189 (2013). 22166267 10.1136/tobaccocontrol-2011-050157 PMC3401240

[bib44] Page, R. L., Slejko, J. F. & Libby, A. M. A citywide smoking ban reduced maternal smoking and risk for preterm births: a Colorado natural experiment. J. Womens Health (Larchmt) 21, 621–627 (2012). 22401497 10.1089/jwh.2011.3305

[bib45] Been, J. V. et al. Impact of smoke-free legislation on perinatal and infant mortality: a national quasi-experimental study. Sci. Rep. 5, 13020 (2015). 26268789 10.1038/srep13020 PMC4534797

[bib46] Shang, C. The effect of smoke-free air law in bars on smoking initiation and relapse among teenagers and young adults. Int. J. Environ. Res. Public Health 12, 504–520 (2015). 25584419 10.3390/ijerph120100504 PMC4306876

[bib47] Wang, Z. et al. Effects of secondhand smoke exposure on asthma morbidity and health care utilization in children: a systematic review and meta-analysis. Ann. Allergy Asthma Immunol. 115, 396–401 (2015). 26411971 10.1016/j.anai.2015.08.005

[bib48] Jones, L. L., Hassanien, A., Cook, D. G., Britton, J. & Leonardi-Bee, J. Parental smoking and the risk of middle ear disease in children: a systematic review and meta-analysis. Arch. Pediatr. Adolesc. Med. 166, 18–27 (2012). 21893640 10.1001/archpediatrics.2011.158

[bib49] Akhtar, P. C., Currie, D. B., Currie, C. E. & Haw, S. J. Changes in child exposure to environmental tobacco smoke (CHETS) study after implementation of smoke-free legislation in Scotland: national cross sectional survey. BMJ 335, 545 (2007). 17827487 10.1136/bmj.39311.550197.AE PMC1976539

[bib50] Holliday, J. C., Moore, G. F. & Moore, L. A. Changes in child exposure to secondhand smoke after implementation of smoke-free legislation in Wales: a repeated cross-sectional study. BMC Public Health 9, 430 (2009). 19930678 10.1186/1471-2458-9-430 PMC2789068

[bib51] Jarvis, M. J., Sims, M., Gilmore, A. & Mindell, J. Impact of smoke-free legislation on children's exposure to secondhand smoke: cotinine data from the Health Survey for England. Tob. Control 21, 18–23 (2012). 21527405 10.1136/tc.2010.041608

[bib52] Moore, G. F., Currie, D., Gilmore, G., Holliday, J. C. & Moore, L. Socioeconomic inequalities in childhood exposure to secondhand smoke before and after smoke-free legislation in three UK countries. J. Public Health (Oxf) 34, 599–608 (2012). 22448041 10.1093/pubmed/fds025 PMC3503469

[bib53] Cheng, K. W., Glantz, S. A. & Lightwood, J. M. Association between smokefree laws and voluntary smokefree-home rules. Am. J. Prev. Med. 41, 566–572 (2011). 22099232 10.1016/j.amepre.2011.08.014 PMC3222862

[bib54] Nazar, G. P. et al. Association between being employed in a smoke-free workplace and living in a smoke-free home: evidence from 15 low and middle income countries. Prev. Med. 59, 47–53 (2014). 24287123 10.1016/j.ypmed.2013.11.017 PMC3898883

[bib55] Lee, J. T., Glantz, S. A. & Millett, C. Effect of smoke-free legislation on adult smoking behaviour in England in the 18 months following implementation. PLoS ONE 6, e20933 (2011). 21698295 10.1371/journal.pone.0020933 PMC3115957

[bib56] Mons, U. et al. Impact of national smoke-free legislation on home smoking bans: findings from the International Tobacco Control Policy Evaluation Project Europe Surveys. Tob. Control 22, e2–e9 (2013). 22331456 10.1136/tobaccocontrol-2011-050131 PMC4010876

[bib57] Moore, G. F. et al. Prevalence of smoking restrictions and child exposure to secondhand smoke in cars and homes: a repeated cross-sectional survey of children aged 10-11 years in Wales. BMJ Open 5, e006914 (2015). 10.1136/bmjopen-2014-006914 PMC4316441 25636793

[bib58] Been, J. V. et al. Effect of smoke-free legislation on perinatal and child health: a systematic review and meta-analysis. Lancet 383, 1549–1560 (2014). 24680633 10.1016/S0140-6736(14)60082-9

[bib59] McKinnon, B., Auger, N. & Kaufman, J. S. The impact of smoke-free legislation on educational differences in birth outcomes. J. Epidemiol. Community Health 69, 937–943 (2015). 25987722 10.1136/jech-2015-205779

[bib60] Been, J. V., Millett, C., Lee, J. T., van Schayck, C. P. & Sheikh, A. Smoke-free legislation and childhood hospitalisations for respiratory tract infections. Eur. Respir. J. 46, 697–706 (2015). 26022951 10.1183/09031936.00014615

[bib61] Lee, S. L., Wong, W. H. & Lau, Y. L. Smoke-free legislation reduces hospital admissions for childhood lower respiratory tract infection. Tob. Control. http://dx.doi.org/10.1136/tobaccocontrol-2015-052541 (2016). 10.1136/tobaccocontrol-2015-052541 26769122

[bib62] Hawkins, S. S., Hristakeva, S., Gottlieb, M. & Baum, C. F. Reduction in emergency department visits for children's asthma, ear infections, and respiratory infections after the introduction of state smoke-free legislation. Prev. Med. 89, 278–285 (2016). 27283094 10.1016/j.ypmed.2016.06.005 PMC8323994

[bib63] Arnott, D. et al. Can the Dutch Government really be abandoning smokers to their fate? Lancet 379, 121–122 (2012). 22169107 10.1016/S0140-6736(11)61855-2

[bib64] Joossens, L. & Raw, M. The Tobacco Control Scale 2013 in Europe [Internet]. Association of European Cancer Leagues; 2014 [cited 7 April 2016]. Available from http://www.europeancancerleagues.org/images/TobaccoControl/TCS_2013_in_Europe_13-03-14_final_1.pdf.

[bib65] Kruize, A., Zimmerman, C. & Bieleman, B. Monitor naleving rookvrije werkplek 2010 [Internet]. Intraval; 2011 [cited 7 April 2016]. Available from http://www.intraval.nl/pdf/MRN10_c42.pdf.

[bib66] ITC Project. ITC Netherlands National Report [Internet]. University of Waterloo; The Hague, The Netherlands: STIVORO (Dutch Expert Centre on Tobacco Control), (2010). Available from http://www.itcproject.org/files/Report_Publications/National_Report/netherlandsnationalreportsingleweb.pdf.

[bib67] Nederlandse Voedsel- en Warenautoriteit. Inventarisatie naleefniveau rookvrije horeca [Internet]. Ministerie van Economische Zaken, (2010). Available from https://www.nvwa.nl/onderwerpen/eten-drinken-roken/dossier/roken-en-tabak/inventarisatie-naleefniveau-rookvrije-horeca-door-intraval.

[bib68] Amaral, M. The Effect of Local Smoking Ordinances on Fetal Development: Evidence from California (University of the Pacific, 2009).

[bib69] Cox, B., Martens, E., Nemery, B., Vangronsveld, J. & Nawrot, T. S. Impact of a stepwise introduction of smoke-free legislation on the rate of preterm births: analysis of routinely collected birth data. BMJ 346, f441 (2013). 23412829 10.1136/bmj.f441 PMC3573179

[bib70] Hade E. Analyses of the Impact of the Ohio Smoke-Free Workplace Act (Ohio Department of Health, 2011). Available from http://www.odh.ohio.gov/~/media/ODH/ASSETS/Files/web%20team/features/reportsonsmokefreeworkplaceact.ashx.

[bib71] Hankins, S. & Tarasenko, Y. Do smoking bans improve neonatal health? Health Serv. Res. http://dx.doi.org/10.1111/1475-6773.12451 (2016). 10.1111/1475-6773.12451 PMC5034216 26841359

[bib72] Hawkins, S. S., Baum, C. F., Oken, E. & Gillman, M. W. Associations of tobacco control policies with birth outcomes. JAMA Pediatr. 168, e142365 (2014). 25365250 10.1001/jamapediatrics.2014.2365 PMC4240616

[bib73] Vicedo-Cabrera, A. M. et al. Benefits of smoking bans on preterm and early-term births: a natural experimental design in Switzerland. Tob. Control. http://dx.doi.org/10.1136/tobaccocontrol-2015-052739 (2016). 10.1136/tobaccocontrol-2015-052739 27118814

[bib74] Been, J. V., Mackenbach, J. P., Millett, C., Basu, S. & Sheikh, A. Tobacco control policies and perinatal and child health: a systematic review and meta-analysis protocol. BMJ Open 5, e008398 (2015). 10.1136/bmjopen-2015-008398 PMC4593151 26399572

[bib75] Markowitz, S. The effectiveness of cigarette regulations in reducing cases of Sudden Infant Death Syndrome. J. Health Econ. 27, 106–133 (2008). 17498829 10.1016/j.jhealeco.2007.03.006

[bib76] King, C., Markowitz, S. & Ross, H. Tobacco control policies and sudden infant death syndrome in developed nations. Health Econ. 24, 1042–1048 (2015). 25044665 10.1002/hec.3090

[bib77] Anandan, C., Nurmatov, U., van Schayck, O. C. & Sheikh, A. Is the prevalence of asthma declining? Systematic review of epidemiological studies. Allergy 65, 152–167 (2010). 19912154 10.1111/j.1398-9995.2009.02244.x

[bib78] Grad, R. & Morgan, W. J. Long-term outcomes of early-onset wheeze and asthma. J. Allergy Clin. Immunol. 130, 299–307 (2012). 22738675 10.1016/j.jaci.2012.05.022 PMC3424262

[bib79] Gaudreau, K., Sanford, C. J., Cheverie, C. & McClure, C. The effect of a smoking ban on hospitalization rates for cardiovascular and respiratory conditions in Prince Edward Island, Canada. PLoS ONE 8, e56102 (2013). 23520450 10.1371/journal.pone.0056102 PMC3592861

[bib80] Mackay, D., Haw, S., Ayres, J. G., Fischbacher, C. & Pell, J. P. Smoke-free legislation and hospitalizations for childhood asthma. N. Engl. J. Med. 363, 1139–1145 (2010). 20843248 10.1056/NEJMoa1002861

[bib81] Millett, C., Lee, J. T., Laverty, A. A., Glantz, S. A. & Majeed, A. Hospital admissions for childhood asthma after smoke-free legislation in England. Pediatrics 131, e495–e501 (2013). 23339216 10.1542/peds.2012-2592 PMC4528337

[bib82] Rayens, M. K. et al. Reduction in asthma-related emergency department visits after implementation of a smoke-free law. J. Allergy Clin. Immunol. 122, 537–541 (2008). 18692884 10.1016/j.jaci.2008.06.029

[bib83] Croghan, I. T. et al. Impact of a countywide smoke-free workplace law on emergency department visits for respiratory diseases: a retrospective cohort study. BMC Pulm. Med. 15, 6 (2015). 25608660 10.1186/1471-2466-15-6 PMC4417313

[bib84] Landers, G. The impact of smoke-free laws on asthma discharges: a multistate analysis. Am. J. Public Health 104, e74–e79 (2014). 10.2105/AJPH.2013.301697 PMC3935700 24328638

[bib85] Been, J. V. et al. Smoke-free legislation and the incidence of paediatric respiratory infections and wheezing/asthma: interrupted time series analyses in the four UK nations. Sci. Rep. 5, 15246 (2015). 26463498 10.1038/srep15246 PMC4604467

[bib86] Dove, M. S., Dockery, D. W. & Connolly, G. N. Smoke-free air laws and asthma prevalence, symptoms, and severity among nonsmoking youth. Pediatrics 127, 102–109 (2011). 21149426 10.1542/peds.2010-1532 PMC3375465

[bib87] Semple, M. G., Taylor-Robinson, D. C., Lane, S. & Smyth, R. L. Household tobacco smoke and admission weight predict severe bronchiolitis in infants independent of deprivation: prospective cohort study. PLoS ONE 6, e22425 (2011). 21811609 10.1371/journal.pone.0022425 PMC3139660

[bib88] Wilson, K. M., Pier, J. C., Wesgate, S. C., Cohen, J. M. & Blumkin, A. K. Secondhand tobacco smoke exposure and severity of influenza in hospitalized children. J. Pediatr. 162, 16–21 (2013). 22863259 10.1016/j.jpeds.2012.06.043

[bib89] Hill, S., Amos, A., Clifford, D. & Platt, S. Impact of tobacco control interventions on socioeconomic inequalities in smoking: review of the evidence. Tob. Control 23, e89–e97 (2014). 24046211 10.1136/tobaccocontrol-2013-051110

[bib90] Diepeveen, S., Ling, T., Suhrcke, M., Roland, M. & Marteau, T. M. Public acceptability of government intervention to change health-related behaviours: a systematic review and narrative synthesis. BMC Public Health 13, 756 (2013). 23947336 10.1186/1471-2458-13-756 PMC3765153

[bib91] Kalkhoran, S. & Glantz, S. A. Smoke-free policies: cleaning the air with money to spare. Lancet 383, 1526–1528 (2014). 24680632 10.1016/S0140-6736(14)60224-5

[bib92] Filippidis, F. T. et al. Relationship of secondhand smoke exposure with sociodemographic factors and smoke-free legislation in the European Union. Eur. J. Public Health 26, 344–349 (2016). 26511601 10.1093/eurpub/ckv204

[bib93] McDaniel, P. A., Smith, E. A. & Malone, R. E. The tobacco endgame: a qualitative review and synthesis. Tob. Control 0, 1–11 (2015). 10.1136/tobaccocontrol-2015-052356 PMC5036259 26320149

[bib94] Harris, J. E., Balsa, A. I. & Triunfo, P. Tobacco control campaign in Uruguay: impact on smoking cessation during pregnancy and birth weight. J. Health Econ. 42, 186–196 (2015). 25985121 10.1016/j.jhealeco.2015.04.002

[bib95] Patrick, S. W., Warner, K. E., Pordes, E. & Davis, M. M. Cigarette tax increase and infant mortality. Pediatrics 137, 1–8 (2016). 10.1542/peds.2015-2901 PMC4702024 26628730

[bib96] Bartholomew, K. S. Policy options to promote smokefree environments for children and adolescents. Curr. Probl. Pediatr. Adolesc. Health Care 45, 146–181 (2015). 26032229 10.1016/j.cppeds.2015.04.001

[bib97] King, B. A., Patel, R., Babb, S. D., Hartman, A. M. & Freeman, A. National and state prevalence of smoke-free rules in homes with and without children and smokers: two decades of progress. Prev. Med. 82, 51–58 (2016). 26601642 10.1016/j.ypmed.2015.11.010 PMC4766981

[bib98] Wise, J. Smoking in cars carrying children will be illegal in England from October. BMJ 350, h836 (2015). 25700694 10.1136/bmj.h836

[bib99] Zhang, X., Martinez-Donate, A. & Rhoads, N. Parental practices and attitudes related to smoke-free rules in homes, cars, and outdoor playgrounds in US households with underage children and smokers, 2010-2011. Prev. Chronic Dis. 12, E96 (2015). 26086609 10.5888/pcd12.140553 PMC4473600

